# Metabolic Cost of the Activation of Immune Response in the Fish-Eating Myotis (*Myotis vivesi*): The Effects of Inflammation and the Acute Phase Response

**DOI:** 10.1371/journal.pone.0164938

**Published:** 2016-10-28

**Authors:** Aída Otálora-Ardila, L. Gerardo Herrera M., José Juan Flores-Martínez, Kenneth C. Welch

**Affiliations:** 1 Posgrado en Ciencias Biológicas, Instituto de Biología, Universidad Nacional Autónoma de México, Ciudad de México, México; 2 Estación de Biología Chamela, Instituto de Biología, Universidad Nacional Autónoma de México, San Patricio, Jalisco, México; 3 Laboratorio de Sistemas de Información Geográfica, Departamento de Zoología, Instituto de Biología, Universidad Nacional Autónoma de México, Ciudad de México, México; 4 Department of Biological Sciences, University of Toronto Scarborough, Toronto, Canada; CSIRO, AUSTRALIA

## Abstract

Inflammation and activation of the acute phase response (APR) are energetically demanding processes that protect against pathogens. Phytohaemagglutinin (PHA) and lipopolysaccharide (LPS) are antigens commonly used to stimulate inflammation and the APR, respectively. We tested the hypothesis that the APR after an LPS challenge was energetically more costly than the inflammatory response after a PHA challenge in the fish-eating Myotis bat (*Myotis vivesi*). We measured resting metabolic rate (RMR) after bats were administered PHA and LPS. We also measured skin temperature (T_skin_) after the LPS challenge and skin swelling after the PHA challenge. Injection of PHA elicited swelling that lasted for several days but changes in RMR and body mass were not significant. LPS injection produced a significant increase in T_skin_ and in RMR, and significant body mass loss. RMR after LPS injection increased by 140–185% and the total cost of the response was 6.50 kJ. Inflammation was an energetically low-cost process but the APR entailed a significant energetic investment. Examination of APR in other bats suggests that the way in which bats deal with infections might not be uniform.

## Introduction

Inflammation and the acute phase response (APR) constitute a series of reactions induced by the vertebrate immune system at the beginning of an infection [1, 2]. Inflammation involves recognition of infection, production of cytokines by activated macrophages and the subsequent recruitment and activation of leukocytes to eliminate the infectious agent [3]. The APR is triggered by different stimuli (e.g. trauma, infection, stress, neoplasia, and inflammation) resulting in a complex systemic reaction characterized by the induction of fever, anorexia, somnolence, lethargy, increased synthesis of hormones and leukocytosis, and production of acute phase proteins [4]. Inflammation and activation of the APR are considered energetically demanding processes [5, 6], and constitute an essential defense against novel pathogens [7]. An increase in energy expenditure to mount an immune response may affect the metabolic rate of an organism [8, 9] thus affecting its energy budget.

Two of the antigens commonly used to stimulate inflammation and the APR are phytohaemagglutinin (PHA) and lipopolysaccharide (LPS), respectively. PHA is a non-pathogenic plant protein and its initial injection induces endothelial permeability, oedema at the site of injection, and infiltration of innate cells and of lymphocytes, the mediators of adaptive immunity [10, 11]. Subsequent injections of PHA produce enhanced inflammation reflecting the induced response of adaptive immunity [12]. LPS is an endotoxin present in most gram-negative bacteria. When injected, LPS stimulates a non-pathogenic, short-lived inflammatory response characterized by the release of proinflammatory cytokines potentially triggering the development of fever, weight loss, and lethargy [13, 14]. Experimental work in homeothermic vertebrates indicates that the effects of PHA and LPS application on metabolic rate are not uniform. For example, PHA administration has been shown to induce large (30%) [15] or small increases in resting metabolic rate (RMR) in some organisms (~5%) [16], no increases in RMR [17–21], and even decreases in RMR in others (-20‒-25%) [18]. Similarly, the effect of LPS administration on vertebrate RMR varies from large (~33‒40%) [22, 23] to small (~10%) [24–26] or null [27, 28]. In theory, LPS administration should evoke a more metabolically expensive response than PHA because it often induces an increase in body temperature (T_b_). Febrile response is thought to increase survival to an immune challenge, but implies a substantial investment of metabolic energy from the host [22]. It has been estimated that a 1°C increase in T_b_ amounts to a 10–15% increase in metabolic rate [29, 30]. However, the above mentioned studies do not permit conclusive comparisons regarding the energetic response elicited by both antigens because most tests involved different species except for house sparrows (*Passer domesticus*) [15, 18, 23].

We tested the hypothesis that the APR after an LPS challenge was energetically more costly than the inflammatory response after a PHA challenge in the fish-eating Myotis bat (*Myotis vivesi*). Bats are natural hosts of a significant number of disease-vectors (e.g. bacteria, protozoa, viruses) [31–34]. In spite of the importance of the activation of the immune response in the regulation of and defense against disease agents, only some aspects of the inflammatory response have been examined in bats [35–38] and information on the metabolic cost associated with APR activation is scant. For example, there is evidence that RMR increases and body mass decreases after Pallas’ long-tongued nectar bat (*Glossophaga soricina*) were administered an LPS challenge (Herrera M. and Cruz-Neto, personal communication). This finding is intriguing since LPS administration in Pallas´s mastiff bat (*Molossus molossus*) elicits loss of body mass but no fever response [38]. We measured RMR before and after PHA and LPS were administered to compare the metabolic costs of the immune response activated by each antigen. We also examined body mass changes to determine if bats lost mass as a result of the administration of PHA and LPS. Finally, we measured T_b_ after the LPS challenge to determine if fever response was present in fish-eating Myotis, and measured skin swelling after the PHA challenge to confirm the development of local inflamatory response.

## Materials and Methods

### Animal care and housing

Individuals of fish-eating Myotis were captured in Partida Norte Island (28°52’30”N, 113°02’17”W) in October 2013 for the PHA challenge and in March 2014 for the LPS challenge. The island is located in the midriff region of the Gulf of California, Mexico, and it holds the largest known colony of fish-eating Myotis [39]. Individuals were maintained in captivity for one to two weeks before experiments. Bats were first maintained in a tent and in an outdoor flight cage (3.4×2.8×1.8 m) where they were fed with shrimp, salmon and mealworms with water supplied *ad libitum*. Individuals were identified through a unique pattern of dots on their torso made by shaving small (0.5×0.5 cm) areas of fur. Mean (± s.e.) ambient temperature was 29.3 ± 1.5°C for all experiment days. This study was carried out in strict accordance with the recommendations and permits approved by Secretaría de Gobernación (#013/13) and from Dirección General de Vida Silvestre (01947/13), Mexico. All sampling procedures and experimental manipulations were approved as part of obtaining this permit. No other approval was required to conduct the study as there is no IACUC/animal ethics board at our institution.

### PHA and LPS injection procedure

#### PHA injection procedure

Bats (5 males, 5 females) were placed in individual respirometry chambers for 2–4 hours during two days before the initiation of data collection to acclimate them to the surroundings and to pump noise. Chambers consisted of 500 ml horizontally oriented plastic cylinders. Unlike most bats, fish-eating Myotis roost in cavities and crevices between and under rocks, typically adopting a horizontal orientation. Thus, the orientation of chambers used during this study permitted a natural roosting posture. Both the inlet and outlet ports entered through the chamber lid and a length of Pharmed tubing was attached to the outlet port, promoting gas mixing. Bats were loosely wrapped in a paper towel while inside the chambers. This provided a comfortable substrate on which the bats rested and helped to prevent the bat from blocking the outlet port tubing. We collected data during four days after the acclimation period. In the second day of data collection we randomly assigned bats to receive an injection of 50 μL of a 3 mg mL^-1^ solution of PHA (L8754, Sigma, Saint-Louis, MO, USA) in phosphate buffered saline (PBS) or PBS only. The PHA dose (6.01 ± 0.35 mg kg^-1^) was selected based on previous trials that induced an inflammatory response in the study bat. Bats were injected PHA and PBS on the right and left foot, respectively, between 8:00 and 9:40 hours. Prior to injection, the skin surrounding the injection site was sterilized with ethanol. Individuals acted as their own controls and were injected seven days later with the same amount of either PHA or PBS following the same protocol. PHA induces skin swelling in the place of injection, hence, we measured the thickness of the injected footpad using a digital micrometer (Mitutuyo #0030447, Japan) immediately before injection and six hours after injection during all days that we obtained respirometry measurements (see below). All measurements were made three times per bat and averaged (S1 File). The standard error of individual measurements ranged from ±0.05 to ±0.17 mm. To determine if the PHA challenge induced body mass reduction, we measured body mass at the beginning and end of trials one day prior to and three days after injection of PHA and PBS (S1 File).

#### LPS injection procedure

Individuals for the LPS challenge were different than those used for the PHA challenge. Seven bats (3 males, 4 females) were acclimated to the respirometry procedures as described above during one day before data collection. We collected data beginning two days after the acclimation period. In the second day of data collection we randomly assigned bats to receive an injection of either 50 μL of a 1 mg mL^-1^ solution of LPS (L2630, Sigma, Saint-Louis, MO, USA) in PBS or PBS only. The LPS dose (1.75 ± 0.06 mg LPS kg^-1^) was similar to the dose used for a bat of similar size to our study species (~2 mg kg^-1^) [37]. Bats were injected subdermally in the back between 7:00 and 8:10 hours. Prior to injection, the skin surrounding the injection site was sterilized with ethanol. Individuals acted as their own controls and were injected seven days later with LPS or PBS following the same protocol. LPS typically produces a pyrogenic (fever-inducing) response; therefore, we measured bat skin temperature (S1 File) using temperature-sensitive radiotransmitters (BD-2CT, Holohil Systems, Ontario, Canada) attached dorsally between the scapulae. Measurement of skin temperature (T_skin_) is considered a good estimator of T_b_ in bats [40]. Increases in the number of pulses emitted by radiotransmitters is indicative of increase in T_skin_ where pulses interval range from ~24‒25 pulses per minute (0°C) to ~36‒38 pulses per minute (40°C). We used R-1000 receivers (Communication Specialists Inc, Orange, CA, USA) to record the number of pulses emitted by the radiotransmitters every two hours during the whole experiment. We used calibration curves provided by the manufacturer for each radiotransmitter to convert pulses into T_skin_. Trusting manufacture´s calibration might lead to errors in T_skin_ ranging from 0.1 to 1.7°C, but any error is expected to be < 0.5°C in the first nine days of operation [39]. We found a mean difference (mean ± s.e.) of 0.2 ± 0.1°C (*n* = 35) between water temperatures reconstructed with radio-transmitters and with a thermometer. Bat T_skin_ detected by radio-transmitters might be slightly affected by changes in ambient temperature, particularly when ambient temperature is low [41]. Therefore, we recorded the temperature in the metabolic chambers during the experiment. Additionally, to determine if LPS challenge induced body mass reduction, we measured body mass at the beginning and end of the trials in the first day of the experiment, and 1 hour prior and 11 hours after PBS or LPS injection in the second day.

### Respirometry and experimental design

We determined RMR by measuring O_2_ consumption (*V*_*O2*_) using flow-through respirometry during the resting phase of bats (S1 File). In both experiments, we measured O_2_ consumption one day before (Day -1) and the day of the immune stimulation with PHA or LPS injection (Day 0). To measure *V*_*O2*_ rates, external air entered into three metabolic chambers (each containing one bat) and one baseline chamber. Excurrent air from chambers was sequentially delivered to flow-through precision water vapour and O_2_ gas analyzers (Field Metabolic System, Sable Systems International, Las Vegas, NV, USA). Air was scrubbed of water vapour by passage through a column of Drierite (W.A. Hammond DRIERITE, Xenia, OH, USA) after passing through the water vapour meter and before the O_2_ analyzer. Flow of air entering in each chamber and the baseline was set at 400–500 ml min^-1^ and was maintained by the mass-flow controller of the FoxBox.

In both PHA and LPS experiments, each individual was placed in the chamber one hour before its recordings started and maintained there until the end of the experiment. The day of the injection, bats were placed in the chamber immediately after they were administered PHA, LPS or PBS. In the PHA experiment, we recorded from 9:00 to 16:00 hours each day. Our set-up allowed us to record *V*_*O2*_ from six bats per day. Each recording sequence began with measuring *V*_*O2*_ at 1 s interval during 5 minutes from the baseline chamber, followed by 15 minutes from metabolic chamber containing a bat and then another 5 minutes of baseline. By the end of the trial, we had acquired 15-minute recordings per bat corresponding to hours -23, -21, -18, 1, 3, 6, 25, 27, 30, 49, 51 and 54 in relation to PHA or PBS injection. For LPS experiment, we recorded from 8:00 to 19:40 hours. Each recording sequence began with measuring *V*_*O2*_ at 1 s interval during 5 minutes of baseline chamber air, followed by 30 minutes from metabolic chamber containing a bat and then another 5 minutes of baseline. By the end of the trial, we had acquired 30 minute recordings per bat corresponding to hours -21, -18, -15, -13, 1, 3, 5, 7, 9 and 11 in relation to LPS or PBS injection.

For the two experiments the outputs from the flow rates, temperature, and oxygen analyzer were digitized using a Universal Interface II (Sable Systems, Las Vegas, NV, USA) and recorded on a computer using ExpeData acquisition software (v. 1.7.2, Sable Systems International, Las Vegas, NV, USA). Oxygen concentration was converted to ml of gas by application of standard equations [42] assuming a RER of 0.77, which was the mean RER observed in fasted fish-eating Myotis examined in a previous study [43]. *V*_*O2*_ at each hour after PHA or LPS injection was calculated as the lowest five-min mean value of instantaneous oxygen consumption. Metabolic rates were expressed as ml O_2_ h^−1^.

When the effect of treatment on RMR was significant, we calculated the net metabolic cost of the injection response by subtracting the final pre-injection control values for *V*_*O2*_ from each post-injection. Control-corrected *V*_*O2*_ values were converted to their oxy-joules equivalents (*MR*_*kj*_ in kJ hr^-1^) according to the following equation from [40] and assuming the respiratory exchange ratio ($RER=V_{{CO}_{2}}/V_{O_{2}}$; where V_CO2_ is carbon dioxide production rate) was equal to 0.77, which was the average RER value observed in fasted fish-eating Myotis examined in a separate study [43]: *MR*_*kj*_ *= V*_*O2*_ x [16 + 5.164 (RER)]

Following this, we fitted a spline function to these corrected post-injection measurements and calculated the area under the curve using the “rollapply” function in the “zoo” package [44] in R (V. 3.1.0) [45].

### Data analysis

We employed 2-way repeated measures analysis of variance (RM-ANOVA) to test the effect of immune challenges on RMR (*V*_*O2*_), T_skin_, chamber temperature, footpad thickness and body mass. For chamber temperature, we compared only the values for the day of LPS and PBS injections. For body mass, we compared the difference between final and initial body mass one day previous to injection and on the day of the injection. The models examined the effect of treatment (LPS or PHA and PBS), time with respect to injection, and the interaction between these two factors. The assumption of sphericity was examined with Mauchly tests and we applied a Greenhouse-Geisser (G-G) correction when it was not met. We only included data from 5 individuals in the RM-ANOVA corresponding to the LPS challenge due to missing RMR values in two individuals in hours -17, -15, -13 and 1 for the LPS injection, and -15 and 3 for the PBS injection. In the case of RMR, we performed additional tests examining mass-specific post-injection values (*V*_*O2*_/body mass) for the PHA and LPS treatments. We used the mean of body mass values measured at the beginning and end of each trial to estimate mass-specific values. When the factors or their interactions were significant, we conducted post-hoc pairwise comparisons using Tukey´s HSD tests. Net metabolic costs of PBS and LPS injection response were compared to 0 using one-sample *t* tests. All analyses were carried out in Statistica 7 [46]. Values are expressed as mean ± s.e. Statistical significance was considered at *p* ≤ 0.05.

## Results

### Swelling response after PHA immune challenge

**Fig 1:**
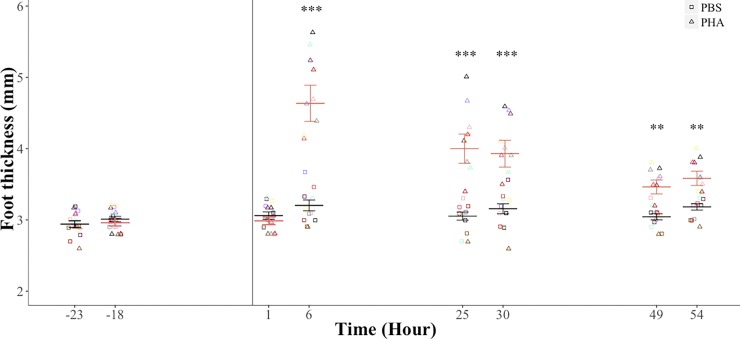
Footpad thickness of the fish-eating Myotis (*M*. *vivesi*) after a PHA immune challenge. We present repeated values for each individual (*n* = 10). Black and pink bars are mean ± standard error for bats on the PBS and PHA treatments, respectively. Vertical line indicates time of injection. * *p* ≤ 0.05, *** *p* = 0.0001.

There was no significant difference in footpad thickness before injection of PHA or PBS (injection treatment: F_1, 9_ = 0.20, *p* = 0.66; time before injection: F_1, 8_ = 1.43, *p* = 0.26; treatment-time interaction: F_1, 9_ = 1.59, *p* = 0.23). Footpad thickness was significantly higher after the injection of PHA (3.7 ± 0.1 mm) than of PBS (3.1 ± 0.1 mm; F_1, 9_ = 32.28, *p* = 0.0001). Time after injection (G-G ε = 0.53, F_2.6, 24.0.4,_ = 22.92, *p* < 0.0001) and the treatment-time interaction (G-G ε = 0.47, F_2.3, 21.4_ = 18.33, *p* < 0.0001) had a significant effect on footpad thickness. Footpad thickness was significantly different between PHA and PBS treatments at 6 (*p* < 0.0001), 25 (*p* < 0.0001), 30 (*p* < 0.0001), 49 (*p* = 0.001) and 54 (*p* = 0.001) hours after the injection (Fig 1).

### Heterotermy in the LPS immune challenge

**Fig 2:**
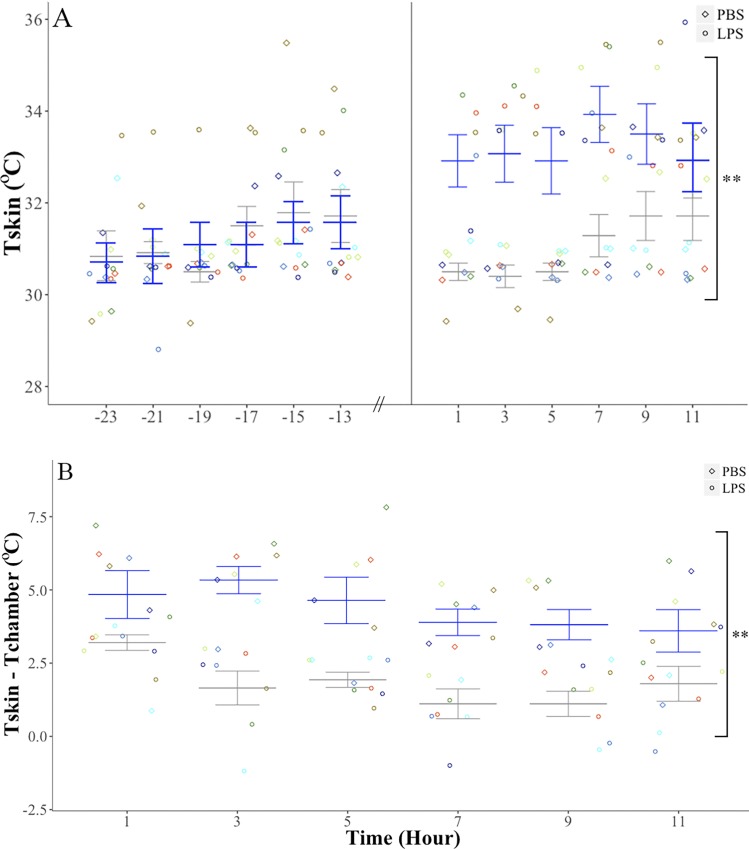
Body temperature in the fish-eating Myotis (*M*. *vivesi*) after a LPS immune challenge. A) Skin temperature (T_skin_) before and after PBS or LPS injection. B) Difference between skin and ambient temperatures (T_chamber_) after PBS ot LPS injection. We present repeated values for each individual (*n* = 7). Gray and blue bars are mean ± standard error for bats on the PBS and LPS treatments, respectively. The vertical line indicates time of injection. ** *p* ≤ 0.01.

Body temperature before the injection did not differ among bats when assigned to the PBS (31.0 ± 0.4°C) or LPS treatments (30.8 ± 0.5°C; injection treatment: F_1, 6_ = 0.06, *p* = 0.81). The effect of time before injection was significant (G-G ε = 0.38, F_1.5, 9.2_ = 6.06, *p* = 0.02) but the treatment-time interaction was not (G-G ε = 0.39, F_1.5, 9.4_ = 1.09, *p* = 0.35). Body temperature was significantly higher after the injection of LPS (33.2 ± 0.6°C) than of PBS (30.8± 0.2°C; F_1, 6_ = 13.74, *p* = 0.01; Fig 2). Although T_skin_ increased with time after the injection (F_5, 30_ = 4.52, *p* = 0.003), the treatment-time interaction was not significant (F_5, 30_ = 1.87, *p* = 0.12). The temperature of the chambers was not significantly different between treatments (F_1, 6_ = 0.1, *p* = 0.7; PHA: 29.1 ± 0.2°C, LPS: 28.9 ± 0.2°C) or as a function of the treatment-time interaction (F_5, 30_ = 0.4, *p* = 0.8), although it varied with time of the day (F_5, 30_ = 155.4, *p* < 0.0001): the largest fluctuation in chamber temperature occurred between that recorded one hour after injection (PHA: 27.3 ± 0.2°C, LPS: 27.1 ± 0.3°C) and that recorded 9 hours after injection (PHA: 30.6 ± 0.3°, LPS: 30.1 ± 0.3°). The difference between T_skin_ and the temperature of the chamber was significantly higher after the injection of LPS (4.3 ± 0.5°C) than of PBS (1.8 ± 0.2°C; F_1, 6_ = 57.15, *p* < 0.0001). Although the difference between bat and chamber temperature decreased with time after injection (F_5, 30_ = 3.06, *p* = 0.02), the higher values found for bats on the LPS treatment were independent of the time elapsed after its injection (treatment-time interaction: F_5, 30_ = 1.39, *p* = 0.25). Peak difference between bat and chamber temperatures was higher after the injection of LPS (5.9 ± 0.3°C, minimum‒maximum range: 4.6‒7.2°C) than of PBS (3.4 ± 0.1°C, 2.9‒4.1°C; F_1, 6_ = 76.23, *p* < 0.001).

### Effects of PHA and LPS immune challenge on body mass

**Fig 3:**
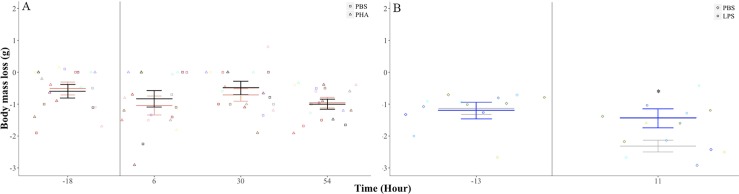
Body mass loss in the fish-eating Myotis (*M*. *vivesi*) due to the PHA and LPS challenges. (A) Bats subjected to a PHA challenge. Black and pink bars are mean ±error for bats on the PBS and PHA treatments, respectively (B) Bats subjected to a LPS challenge. Gray and blue bars are mean ± standard error for bats on the PBS and LPS treatments, respectively. We present repeated values for each individual for the PHA (*n* = 10) and LPS (*n* = 7) challenges. Vertical lines indicate time of injection. * *p* ≤ 0.05.

There were no significant difference in body mass change before (F_1, 9_ = 0.20, *p* = 0.65) and after the injection of PBS or PHA (injection treatment: F_1, 9_ = 0.26, *p* = 0.61; time after injection: F_2, 18_ = 2.60, *p* = 0.10; treatment-time interaction: F_2, 18_ = 0.23, *p* = 0.79; Fig 3A). There was no difference in body mass change before the injection of PBS or LPS (F_1, 6_ = 1.85, *p* = 0.22) but bats lost more body mass after the injection of LPS (-2.3 ± 0.2 g) than PBS (-1.4 ± 0.3 g; F_1, 6_ = 5.21, *p* = 0.05; Fig 3B). Bats lost 7.9 ± 0.01% and 5.2 ± 0.01% of initial body mass after the LPS and the PBS injections, respectively.

### Effects of PHA and LPS immune challenge on RMR

**Fig 4:**
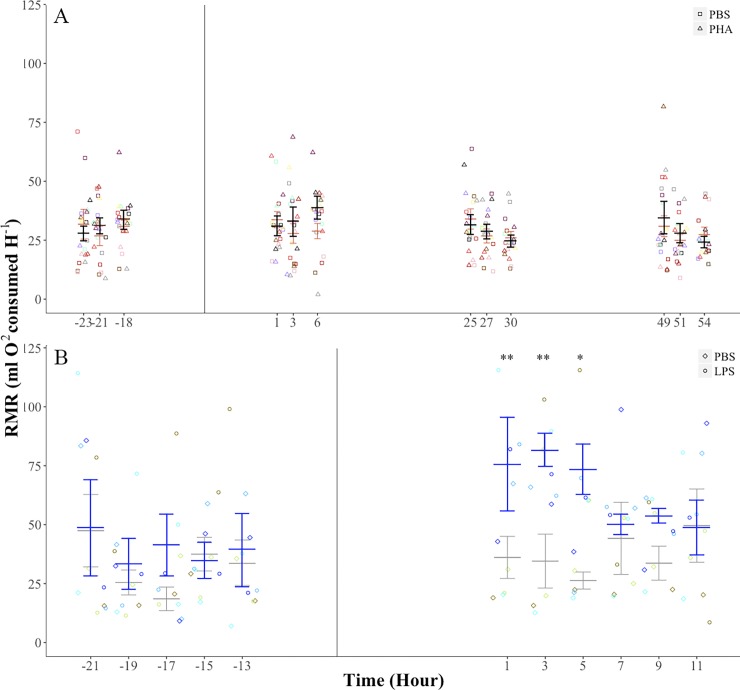
Resting metabolic rate (RMR) of the fish-eating Myotis (*M*. *vivesi*) as a function of PHA and LPS challenges. (A) PHA challenge. Black and pink bars are mean ± error for bats on the PBS and PHA treatments, respectively. (B) LPS challenge. Gray and blue bars are mean ± standard error for bats on the PBS and LPS treatments, respectively. We present repeated values for each individual for the PHA (*n* = 10) and LPS (*n* = 5) challenges.Vertical lines indicates time of injection. * *p* ≤ 0.05, ** *p* = 0.01.

For bats on the PHA treatment, there were no significant differences in RMR prior to the injection of PHA or PBS (injection treatment: F_1, 9_ = 0.06, *p* = 0.80; time before injection: F_2, 18_ = 1.26, *p* = 0.30; treatment-time interaction: F_2, 18_ = 1.08, *p* = 0.35; Fig 4A). After the injection of PHA or PBS, the effects of injection treatment (F_1, 9_ = 1.12, *p* = 0.31), time after injection (F_5, 45_ = 1.98, *p* = 0.10) and the treatment-time interaction (G-G ε = 0.38, F_31.9, 17.2,_ = 0.85, *p* = 0.43) on RMR were not significant (Fig 4A). Mass-specific RMR values were also similar following injection of PBS or PHA (injection treatment: F_1, 6_ = 2.89, *p* = 0.13; time after injection: F_5, 30_ = 1.56, *p* = 0.19; treatment-time interaction: F_5, 30_ = 1.58, *p* = 0.19). For bats in the LPS experiment, there were no significant differences in RMR prior to the injection of LPS or PBS (injection treatment: F_1, 4_ = 0.15, *p* = 0.71; time before injection: F_4, 16_ = 2.64, *p* = 0.07; treatment-time interaction: F_4, 16_ = 0.74, *p* = 0.57; Fig 4B). After the injection of LPS or PBS, the effects of the injection treatment (F_1, 4_ = 5.38, *p* = 0.08) and of time after the injection (F_5, 20_ = 1.07, *p* = 0.40) on RMR were not significant but the treatment-time interaction (F_5, 20_ = 3.58, *p* = 0.01) was significant. Pairwise post-hoc comparisons showed significant differences in RMR between LPS and PBS treatments only at 1 (*p* = 0.01), 3 (*p* = 0.03), and 5 (*p* = 0.03) hours after the injection (Fig 4B). Mass-specific RMR values post-injection of PBS and LPS showed a similar pattern (injection treatment: F_1, 4_ = 5.14, *p* = 0.08; time after injection: F_5, 20_ = 1.02, *p* = 0.42; treatment-time interaction: F_5, 20_ = 3.64, *p* = 0.01; LPS vs PBS: *p*_hour 1_ = 0.02, *p*_hour 3_ = 0.04, *p*_hour 5_ = 0.04). The total cost of the response to PBS injection was not significantly different from 0 (0.90 ±1.05 kJ; *t*_6_ = 0.8, *p* = 0.4) but it was significantly greater (6.50 ± 0.70 kJ; *t*_6_ = 9.2, *p* < 0.0001) after LPS injection.

## Discussion

We observed contrasting metabolic responses in fish-eating Myotis exposed to PHA and LPS challenges. Injection of PHA elicited swelling of the footpad that lasted for several days but RMR did not increase significantly and body mass loss was similar to that when bats were injected PBS. In contrast, LPS injection produced a significant increase in T_skin_ and in RMR and body mass loss was higher than when bats were injected with PBS.

The caloric cost of PHA challenge has been measured in other vertebrates but doses applied vary. Lack of a significant increase in RMR in fish-eating Myotis after a PHA challenge is similar to what has been found in other mammals. For example, RMR following PHA administration did not increase in white-footed mouse (*Peromyscus leucopus*, 3.24 mg kg^-1^) [17], Mongolian gerbils (*Meriones unguiculatus*, 2.04‒3.20 mg kg^-1^) [19] or tuco-tucos (*Ctenomys talarum*, ~1 mg kg^-1^) [20]. Our findings are also similar to those found in some birds, including the lesser kestrel (*Falco naumanni*, 1.83 mg kg^-1^) [21], house sparrow (*Passer domesticus*, 3.70 mg kg^-1^) [18], and Japanese quail (*Coturnix coturnix*, 0.49‒0.59 mg kg^-1^) [47] suggesting that the inflammatory response is not an energetically costly process. In fact, only two studies have proven that inflammation might elicit a significant metabolic cost: RMR increased to a large extent (~30%) in house sparrow (3.70 mg kg^-1^) [15] and to a moderate extent (~5%) in the great tit (*Parus major*, 5.71 mg kg^-1^) [18]. Furthermore, heterogeneity of the metabolic response to PHA injection is evidenced by a reported reduction (-20‒-25%) in RMR in the tree sparrow (*Passer montanus*) [18]. The contrasting results found in two populations of house sparrow [15, 18] illustrate the difficulty in establishing generalizations regarding the metabolic cost of inflammation. Swelling responses vary seasonally in populations of house sparrows and this variation matches the metabolic responses elicited by studies conducted at different times of the year [18]. This observation might have implications for the interpretation of our findings in fish-eating Myotis because the magnitude of swelling varies seasonally in this species (Otálora-Ardila and Herrera M, personal communication) and in congeners (greater mouse-eared bat *M*. *myotis*) [35]. Specifically, swelling after a PHA challenge in free-ranging non-reproductive females of fish-eating Myotis is lower in spring and autumn than in winter and summer (Otálora-Ardila and Herrera M, personal communication). Swelling measurements reported here were obtained in spring and autumn. Thus it remains to be confirmed if the energetic cost of inflammation is persistently inexpensive throughout the year for fish-eating Myotis. The low energetic cost of inflammation might also be related to the bats life history. Bats are long-lived individuals and their investment in innate immunity should be low, as predicted for animals that have a slow-paced life style [48]. Furthermore, fish-eating Myotis roost under rocks which might expose them to injuries at a higher rate than bats that roost in caves or trees, and this scenario could have resulted in a low-cost inflammatory process as suggested for subterranean rodents [20].

The effect of LPS administration on the RMR of fish-eating Myotis was comparatively great: mean RMR after LPS injection increased by 140–185% with respect to mean RMR measured after PBS injection during the period when this measurement differed between both treatments (1, 3 and 5 hours after injection). Although our study is not strictly comparable to previous studies in vertebrates because mass-specific doses differ, this increase in metabolic rate was much higher than in wild and model vertebrates. RMR increased by ~33–40% in Pekin ducks (*Anas platyrhynchos*; 0.1 mg kg^-1^) and house sparrows (5 mg kg^-1^) after an LPS challenge [22, 23], and the increase was modest (~10%) in lab rats (*Rattus norvegicus*; 0.05 mg kg^-1^) [25] and null in lab mice (*Mus musculus*; 0.5 mg kg^-1^) [27]. Therefore, with the exception of house sparrows [23], higher increments of RMR in our study compared to other studies [22, 25, 27] might be the result of our use of a relatively higher LPS dose. The increase in RMR after the LPS injection amounted to an average total increased energy cost of 6.50 kJ, but daily energy requirements have not been measured for fish-eating Myotis, making interpretation of the significance of this additional energy burden more difficult. Initial body mass of fish-eating Myotis during the LPS challenge ranged from 24.5 to 32.0 g and daily energy requirements predicted by a published allometric scaling relationship between DEE and body mass in mammals [Log_e_ FMR (kJ day^-1^) = 1.871 + 0.670 ∙ Log_e_ body mass (g)] [49] range from 55 to 66 kJ]. If we assume a similar energy budget for fish-eating Myotis, the average cost of immune activation after an LPS challenge equates to ~9.8‒11.8% of its daily energy requirements. The increase in metabolic rate appears to be driven by a parallel increase in body temperature and is reflected in significantly greater decreases in body mass. Mean T_skin_ was 1.4‒3.6°C higher after LPS administration than when the bats were injected PBS. When bats were injected PBS, they appear to remain torpid throughout the experiment with T_skin_ values ranging from 29.4 to 31.7°C. In support of this, with the exception of the measurement 1 hour after the injection, average T_skin_ values in these bats were only 1.6‒1.9°C greater than the chamber temperature, mirroring the pattern found in torpid fish-eating Myotis under captive conditions [50]. When bats were injected LPS, their mean T_skin_ values ranged from 32.0 to 33.9°C and were 3.6 to 5.3°C higher than chamber temperature, although this difference peaked to ~6‒8°C in some instances. We hypothesize that these bats were normothermic for most of the post-LPS injection period because their T_skin_ values are similar to the body temperature of captive thermoregulating fish-eating Myotis [50]. Interestingly, the mean T_skin_ change did not parallel the mean increase in RMR 1 hour after LPS injection. This may be attributable to high variance in T_skin_ values observed at this time point due to the unusually low T_skin_ (26.5°C) recorded in one individual that was almost identical to the temperature of its chamber (25.6°C) indicating that it was torpid. In particular, the increase in T_skin_ temperature is different from the lack of change in T_b_ previously reported for Pallas´s mastiff bats challenged with a higher dose of LPS (4.53 mg kg^-1^) [38]. T_b_ in Pallas´s mastiff bats after LPS administration was similar to the T_skin_ recorded in fish-eating Myotis injected with PBS. Interestingly, body mass loss in fish-eating Myotis after 11 hours of LPS administration (~8%) was similar to the loss in Pallas´s mastiff bats and short-tailed fruit bats after 24 hours of being treated treated with LPS (~7–8%) [37, 38]. In contrast to Pallas´s mastiff bats in which thermosensitive tags were implanted subcutaneously to measure T_b_, our measurements relied on external radiotransmitters that determine T_skin_. However, we are confident that our T_skin_ measurements are an accurate approximation of T_b_ as previously shown in bats [40]. Furthermore, ambient temperatures did not differ between treatments, indicating that higher T_skin_ values detected after LPS truly reflect changes in bat´s T_b_. The pattern of T_skin_ values after the PBS injection mirrored the pattern in T_skin_ values the day before the injection indicating that the injection of the saline did not induce changes in T_b_. In contrast, when the pattern after the LPS injection is compared with the pattern the day before the injection it is clear that an increase in T_b_ occurs associated to the immune challenge.

Bats are natural hosts of an important number of disease-vectors [31–34] but information on the energetic cost of activating defenses against these challenges is scant. For example, grooming is one of the first lines of defenses used by bats against ectoparasites that might carry infectious diseases and this behavior might increase metabolic rate and led to body mass loss if sustained at a high rate [51]. Once infected, bats might resort to initial immune responses via inflammation and/or fever. We found that inflammation is an energetically low-cost process but that fever entails a significant increase in metabolic rate. Studying the metabolic aspects of activating the bat immune system is a promising research area considering the large taxonomical and ecological diversity of this order. For example, the contrasting findings in our study and that with Pallas´s mastiff bats in relation to changes in T_b_ as part of the APR suggests that the way in which bats deal with infections is not uniform.

## Supporting Information

S1 File Data for individual bats challenged with PHA or LPS. Body mass and resting metabolic rate is presented for individuals before and after the injection of the antigen (PHA or LPS) and its control (PBS). Foot thickness data is presented for bats challenged with PHA. Skin temperature data is presented for bats challenged with LPS.
(CSV)

## References

[1] Owen-Ashley NT, Wingfield JC. Acute phase responses of passerine birds: characterization and seasonal variation. J Ornithol. 2007;148: 583–591. 10.1007/s10336-007-0197-2

[2] Viljoen H, Bennett NC, Lutermann H. Life-history traits, but not season, affect the febrile response to a lipopolysaccharide challenge in highveld mole-rats. J Zool. 2011;285: 222–229. 10.1111/j.1469-7998.2011.00833.x

[3] Barton GM. A calculated response: control of inflammation by the innate immune system. J Clin Invest. 2008;118: 413–420. 10.1172/JCI34431 18246191 PMC2214713

[4] Baumann H, Gauldie J. The acute phase response. Immunol Today. 1994;15: 74–80. 10.1016/0167-5699(94)90137-6 7512342

[5] Lochmiller RL, Deerenberg C. Trade-offs in evolutionary immunology: just what is the cost of immunity? Oikos. 2000;88: 87–98.

[6] Klasing K. The cost of immunity. Curr Zool. 2004;50: 961–969.

[7] Buehler DM, Tieleman BI, Piersma T. How do migratory species stay healthy over the annual cycle? A conceptual model for immune function and for resistance to disease. Integr Comp Biol. 2010;50: 346–357. 10.1093/icb/icq055 21558209

[8] Sheldon BC, Verhulst S. Ecological immunology: costly parasite defences and trade-offs in evolutionary ecology. Trends Ecol Evol. 1996;11: 317–321. 21237861 10.1016/0169-5347(96)10039-2

[9] Hasselquist D, Nilsson J-Å. Physiological mechanisms mediating costs of immune responses: what can we learn from studies of birds? Anim Behav. 2012;83: 1303–1312. 10.1016/j.anbehav.2012.03.025

[10] Kennedy M, Nager R. The perils and prospects of using phytohaemagglutinin in evolutionary ecology. Trends Ecol Evol. 2006;21: 653–655. 10.1016/j.tree.2006.09.017 17028055

[11] Boughton RK, Joop G, Armitage SAO. Outdoor immunology: methodological considerations for ecologists: advancing ecological immunology methods. Funct Ecol. 2011;25: 81–100. 10.1111/j.1365-2435.2010.01817.x

[12] Demas G, Greives T, Chester E, French S. The energetics of immunity: mechanisms of trade-off in immunology In: Demas G., Nelson R., editors. Ecoimmunology. Oxford: Oxford University Press; 2011 pp. 259–296.

[13] Bonneaud C, Mazuc J, Gonzalez G, Haussy C, Chastel O, Faivre B, et al Assessing the cost of mounting an immune response. Am Nat. 2003;161: 367–379. 10.1086/346134 12703483

[14] Canale CI, Henry P-Y. Energetic costs of the immune response and torpor use in a primate. Funct Ecol. 2011;25: 557–565. 10.1111/j.1365-2435.2010.01815.x

[15] Martin LB, Scheuerlein A, Wikelski M. Immune activity elevates energy expenditure of house sparrows: a link between direct and indirect costs? Proc R Soc B Biol Sci. 2003;270: 153–158. 10.1098/rspb.2002.2185 12590753 PMC1691219

[16] Nilsson J-Å, Granbom M, Råberg L. Does the strength of an immune response reflect its energetic cost? J Avian Biol. 2007;38: 488–494. 10.1111/j.2007.0908-8857.03919.x

[17] Derting TL, Compton S. Immune response, not immune maintenance, is energetically costly in wild white‐footed mice (*Peromyscus leucopus*). Physiol Biochem Zool. 2003;76: 744–752. 10.1086/375662 14671721

[18] Lee KA, Martin LB, Wikelski MC. Responding to inflammatory challenges is less costly for a successful avian invader, the house sparrow (*Passer domesticus*), than its less-invasive congener. Oecologia. 2005;145: 243–250. 10.1007/s00442-005-0113-5 15965757

[19] Zhiquiang Z, Fengtong Q, Dehua W. Sex and seasonal differences and energetic cost of phytohemagglutinin responses in wild Mongolian gerbils (*Meriones unguiculatus*). Acta Theriol Sin. 2011;3: 284–290.

[20] Merlo JL, Cutrera AP, Luna F, Zenuto RR. PHA-induced inflammation is not energetically costly in the subterranean rodent *Ctenomys talarum* (tuco-tucos). Comp Biochem Physiol A Mol Integr Physiol. 2014;175: 90–95. 10.1016/j.cbpa.2014.05.021 24905647

[21] Rodríguez A, Broggi J, Alcaide M, Negro JJ, Figuerola J. Determinants and short-term physiological consequences of PHA immune response in lesser kestrel nestlings. J Exp Zool Part Ecol Genet Physiol. 2014;321: 376–386. 10.1002/jez.1868 24807828

[22] Marais M, Maloney SK, Gray DA. The metabolic cost of fever in Pekin ducks. J Therm Biol. 2011;36: 116–120. 10.1016/j.jtherbio.2010.12.004

[23] King MO, Swanson DL. Activation of the immune system incurs energetic costs but has no effect on the thermogenic performance of house sparrows during acute cold challenge. J Exp Biol. 2013;216: 2097–2102. 10.1242/jeb.079574 23430994

[24] Burness G, Armstrong C, Fee T, Tilman-Schindel E. Is there an energetic-based trade-off between thermoregulation and the acute phase response in zebra finches? J Exp Biol. 2010;213: 1386–1394. 10.1242/jeb.027011 20348351

[25] MacDonald L, Begg D, Weisinger RS, Kent S. Calorie restricted rats do not increase metabolic rate post-LPS, but do seek out warmer ambient temperatures to behaviourally induce a fever. Physiol Behav. 2012;107: 762–772. 10.1016/j.physbeh.2012.06.009 22722100

[26] Hegemann A, Matson KD, Versteegh MA, Tieleman BI. Wild skylarks seasonally modulate energy budgets but maintain energetically costly inflammatory immune responses throughout the annual cycle. Powell J, editor. PLoS ONE. 2012;7: e36358 10.1371/journal.pone.0036358 22570706 PMC3343055

[27] Baze MM, Hunter K, Hayes JP. Chronic hypoxia stimulates an enhanced response to immune challenge without evidence of an energetic tradeoff. J Exp Biol. 2011;214: 3255–3268. 10.1242/jeb.054544 21900473

[28] Sköld-Chiriac S, Nord A, Nilsson J-Å, Hasselquist D. Physiological and behavioral responses to an acute-phase response in zebra finches: immediate and short-term effects. Physiol Biochem Zool. 2014;87: 288–298. 10.1086/674789 24642546

[29] Banet M. Fever and survival in the rat. Metabolic versus temperature response. Experientia. 1981;37: 1302–1304. 7327237 10.1007/BF01948375

[30] Kluger MJ. Is fever beneficial? Yale J Biol Med. 1986;59: 89 3488621 PMC2590120

[31] Calisher CH, Childs JE, Field HE, Holmes KV, Schountz T. Bats: important reservoir hosts of emerging viruses. Clin Microbiol Rev. 2006;19: 531–545. 10.1128/CMR.00017-06 16847084 PMC1539106

[32] Luis AD, Hayman DTS, O’Shea TJ, Cryan PM, Gilbert AT, Pulliam JRC, et al A comparison of bats and rodents as reservoirs of zoonotic viruses: are bats special? Proc R Soc B Biol Sci. 2013;280: 20122753–20122753. 10.1098/rspb.2012.2753 23378666 PMC3574368

[33] Mühldorfer K. Bats and bacterial pathogens: a review. Zoonoses Public Health, 2013;60: 93–103. 10.1111/j.1863-2378.2012.01536.x 22862791

[34] Brook CE, Dobson AP. Bats as ‘special’reservoirs for emerging zoonotic pathogens. Trends Microbiol. 2015;23: 172–180. 10.1016/j.tim.2014.12.004 25572882 PMC7126622

[35] Christe P, Arlettaz R, Vogel P. Variation in intensity of a parasitic mite (*Spinturnix myoti*) in relation to the reproductive cycle and immunocompetence of its bat host (*Myotis myotis*). Ecol Lett. 2000;3: 207–212.

[36] Allen LC, Turmelle AS, Mendonça MT, Navara KJ, Kunz TH, McCracken GF. Roosting ecology and variation in adaptive and innate immune system function in the Brazilian free-tailed bat (*Tadarida brasiliensis*). J Comp Physiol B. 2009;179: 315–323. 10.1007/s00360-008-0315-3 19002470 PMC7087743

[37] Schneeberger K, Czirjak GA, Voigt CC. Inflammatory challenge increases measures of oxidative stress in a free-ranging, long-lived mammal. J Exp Biol. 2013;216: 4514–4519. 10.1242/jeb.090837 24031067

[38] Stockmaier S, Dechmann DKN, Page RA, O’Mara MT. No fever and leucocytosis in response to a lipopolysaccharide challenge in an insectivorous bat. Biol Lett. 2015;11: 20150576 10.1098/rsbl.2015.0576 26333664 PMC4614434

[39] Flores-Martinez JJ, Floyd CH, Herrera M. LG, May B. Genetic variation and population size of the endangered fishing bat, *Myotis vivesi*, in Isla Partida In: Sánchez-Cordero V, Medellín R, editors. Contribuciones mastozoológicas en homenaje a Bernardo Villa. México: Universidad Nacional Autónoma de México, Comisión Nacional para el Conocimiento y Uso de la Biodiversidad; 2005 pp. 187–192.

[40] Barclay RMR, Kalcounis MC, Crampton LH, Stefan C, Vonhof MJ, Wilkinson L, et al Can external radiotransmitters be used to assess body temperature and torpor in bats? J Mammal. 1996;77: 1102–1106.

[41] Williams JB, Tieleman BI, Shobrak M. Validation of temperature-sensitive radio transmitters for measurement of body temperature in small animals. Ardea. 2009;97: 120–124. 10.5253/078.097.0115

[42] Lighton JRB. Measuring metabolic rates: a manual for scientists Oxford; New York: Oxford University Press; 2008.

[43] Welch K Jr, Otálora-Ardila A, Herrera M LG, Flores-Martínez JJ. The cost of digestion in the fish-eating myotis (*Myotis vivesi*). J Exp Biol. 2015;218: 1180–1187. 10.1242/jeb.115964 25911733

[44] Zeileis A, Grothendieck G. zoo: S3 Infrastructure for regular and irregular time series. J Stat Softw. 2005;14: 1–27.

[45] R Core Team. R: A Language and environment for statistical computing R Foundation for Statistical Computing, Vienna, Austria 2013.

[46] StatSoft, Inc. Statistica. Version 7 [computer program]. StatSoft, Inc., Tulsa, Okla 2004.

[47] Boughton RK, Bridge ES, Schoech SJ. Energetic trade-offs between immunity and reproduction in male japanese quail (*Coturnix coturnix*). J Exp Zool Part A Ecol Genet Physiol. 2007;307A: 479–487. 10.1002/jez.402 17647272

[48] Previtali MA, Ostfeld RS, Keesing F, Jolles AE, Hanselmann R, Martin LB. Relationship between pace of life and immune responses in wild rodents. Oikos 2012;121: 1483–1492.

[49] Speakman JR, Król E. Maximal heat dissipation capacity and hyperthermia risk: neglected key factors in the ecology of endotherms. J Anim Ecol. 2010;79: 726–746. 10.1111/j.1365-2656.2010.01689.x 20443992

[50] Carpenter RE. Salt and water metabolism in the marine fish-eating bat *Pizonyx vivesi*. Comp Biochem Physiol 1968;24: 951–964.51. 5650499 10.1016/0010-406x(68)90807-4

[51] Giorgi MS, Arlettaz R, Christe P, Vogel P. The energetic grooming costs imposed by a parasitic mite (*Spinturnix myoti*) upon its bat host (*Myotis myotis*). Proc R Soc B Biol Sci. 2001;268: 2071–2075. 10.1098/rspb.2001.1686 11571055 PMC1088850

